# Specimens of Plants

**Published:** 1834

**Authors:** Isaac Colby

**Affiliations:** Curator


### VIII. Specimens of Plants.

The Curator of the Cincinnati Medical Society's Herbarium, in behalf of that Society, returns his grateful acknowledgments to those who have contributed dried specimens of plants, for the commencement of an Herbarium. Its enlargement still depends on the voluntary contributions of individuals; and all who have it in their power to aid in the object, will do the Society a favor, to forward to the Herbarium, such plants as they have in their possession, or can collect.

It would be desirable, so far as may be, that all plants should be collected while in flower; and those which have a succession of flowers, when the first have passed into the early stage of fruit, that the germ may be seen. The herbaceous plants, when not too large, should be taken entire with the root, that the radical leaves may be exhibited; and those which are large, should be accompanied with radical leaves, taken from a plant of younger growth. Many plants cannot have their characters fully exhibited without two or more specimens taken at different stages of frutification. In collecting a plant, it should be the object to exhibit all its diversity of leaves, its flowers both staminate and pistillate, and when it can be, its fruit.

Plants should be dried by absorption between papers, so as to show as near as can be, their natural form and original color. A large quantity of paper, the more the better, should be interposed between them, and a weight of from fifty to eighty pounds applied, and the papers should be changed and well aired every day, till the plants are thoroughly dried. Common wrapping paper does very well.

It would also be desirable that every plant presented to the Herbarium, should be accompanied with a label, containing its locality, time of flowering, habits of growth, the common names, its medical virtues, &c. Plants will, however be very acceptable without any label. Each plant presented, when placed in the Herbarium, will be labelled with its name, the class and order, time of flowering, locality, its medical properties if any, and the name of the donor.

If any persons should have plants whose names they are unable fully to make out, and should be disposed to send their duplicate specimens, numbered, to the Herbarium, accompanied with such remarks respecting the habits of the plants as may occur, the Curatior will take great pleasure in examining them and will, if in his power, return the names attached to the numbers, to those who sent them.

The gentleman in Wheeling, Va. is informed that his unnamed plant is of the genus Euphorbia; but as it was but the part of a plant, and it did not appear certain whether it was annual or perennial, erect or procumbent, the species was not so clearly made out, it is however most probably pilosa—*Euphorbia pilosa.*

For the examination of plants, Nuttall's genera, Torrey's compendium, and Eaton's manual are perhaps the best small works. Nuttall contains nearly all the genera that will be found in Ohio and adjoining states. Torrey, though somewhat deficient in the number of the species, is nevertheless plain and familiar in his descriptions. Eaton contains the fullest catalogue of plants, but has so far simplified his descriptions, that some of the characters necessary to distinguish one species from another, are left out. In addition to these, some elementary work will be necessary for the definition of terms.

ISAAC COLBY, M. D. *Curator.*

